# Illustrirte Vierteljahrsschrift Der Ærztlichen Polytechnick

**Published:** 1878-11

**Authors:** 


Illustrirte Vierteljahrsschrift der Ærztlichen Polytechnick," Bern: J. Dalp's Buchhandlung.

We have received from the publisher the first number of the above journal, which is a decided novelty in medical literature. It purposes to give illustrations and descriptions of all new apparatus used in diagnosis and treatment. Among the appliances and instruments figured in the present number are: Galante's rubber extension apparatus; Badal's optometer; Zaufal's instruments for operating in the nasal passages, and a variety of others.

---

We are indebted to Dr. John T. Nagle, Deputy Register of Records of New York, for a copy of the *City Record*, containing the annual summary of the Bureau of Vital Statistics for 1877. From this valuable report we learn that the total mortality of New York City during last year was 26,203, representing an annual death-rate of 24.50 per thousand. There were 148 suicides, 123 of which were males and 25 females; of the various nationalities, Germany furnished 59, or over one-third.

